# 593 Upper Airway Soot and Facial Burns Do Not Necessitate Intubation

**DOI:** 10.1093/jbcr/iraf019.222

**Published:** 2025-04-01

**Authors:** Jessica Burgess, John Dubensky

**Affiliations:** Eastern Virginia Medical School at Old Dominion University; Macon and Joan Brock Virginia Health Sciences at Old Dominion University

## Abstract

**Introduction:**

Identifying which patients are at risk for respiratory decompensation, whether from airway compromise or inhalation injury, is an important component of initial burn resuscitation. Findings that traditionally suggest the need for intubation include cutaneous burns to the face and neck, soot seen in the upper airway, and carbonaceous sputum. However, evidence suggests that a significant number of pre-burn center intubations are ultimately unnecessary. The purpose of this investigation was to determine if head and neck burns or sputum seen in the upper airways were correlated with the need for intubation.

**Methods:**

This was a retrospective review of adults admitted to our center from 2019-2023 with concern for potential airway compromise or inhalation injury in the setting of a burn or smoke exposure. Patients were deemed as not requiring intubation if they were intubated less than 24 hours (including ICU admission for airway monitoring not ever requiring intubation). Patients were excluded if they died within the first 48 hours of admission. Data collected include burn/exposure mechanism, origin of intubation (in the field, at an outside hospital, or at our center), percent TBSA, presence of face and/or neck burn, soot seen in the upper airway, ventilator days, and grade of inhalation injury seen on bronchoscopy (if performed).

**Results:**

107 patients met inclusion criteria. 60 patients (56.1%) had burns to the face and/or neck (only 3 patients had burns to the neck only without facial involvement). 44 patients (44.9%) had soot seen in the upper airway. A total of 47 patients (44%) remained intubated beyond 24 hours; 60 patients (56.1%) were admitted for airway monitoring, or were extubated in less than 24 hours. Multivariate analysis demonstrated that only percent TBSA was found to have any correlation with the need for intubation beyond 24 hours (ANOVA, P value 0.0022). Neither upper airway soot nor face/neck burns were associated with intubation beyond 24 hours, even when both of these findings were present. These findings were consistent in subgroup analysis of intubated patients only. In the 38 patients admitted to the ICU for airway monitoring, only 2 progressed to requiring intubation; these intubations required one attempt without any need for a surgical airway.

**Conclusions:**

The presence of burns to the face and neck or soot in the upper airway were not correlated with a necessary intubation in our cohort. These signs are unlikely to represent any impending airway compromise, and should not necessitate endotracheal intubation in a patient without other traditional signs of respiratory failure.

**Applicability of Research to Practice:**

To prevent unnecessary intubations, there must be an emphasis on the fact that endotracheal intubation is not required in every patient with upper airway soot or burns to the face and/or neck. Airway monitoring alone is a safe alternative. These points should be incorporated into pre-hospital and emergency medicine education.

**Funding for the Study:**

N/A

